# A polymorphism in the human serotonin 5-HT_2A _receptor gene may protect against systemic sclerosis by reducing platelet aggregation

**DOI:** 10.1186/ar2495

**Published:** 2008-09-01

**Authors:** Lorenzo Beretta, Marta Cossu, Maurizio Marchini, Francesca Cappiello, Andrea Artoni, Giovanna Motta, Raffaella Scorza

**Affiliations:** 1 Referral Center for Systemic Autoimmune Diseases, University of Milan & Fondazione IRCCS Ospedale Maggiore Policlinico, Mangiagalli e Regina Elena, Via Pace 9, 20122 Milan, Italy; 2 A. Bianchi Bonomi Hemophilia and Thrombosis Center, Department of Medicine and Medical Specialties, University of Milan & IRCCS Fondazione Policlinico, Mangiagalli e Regina Elena, Via Pace 9, 20122 Milan, Italy

## Abstract

**Introduction:**

Platelet aggregation may contribute to the pathogenesis of systemic sclerosis: following activation, platelets release significant amounts of serotonin – which promotes vasoconstriction and fibrosis, and further enhances aggregation. The C+1354T polymorphism in the exonic region of the serotonin 2A receptor gene determining the His452Tyr substitution was associated with blunted intracellular responses after serotonin stimulation, and may have a role in susceptibility to scleroderma.

**Methods:**

One hundred and fifteen consecutive systemic sclerosis patients and 140 well-matched healthy control individuals were genotyped by sequence-specific primer-PCR for the His^452^Tyr substitution of the serotonin 2A receptor gene, and associations were sought with scleroderma and its main clinical features. The functional relevance of the His^452^Tyr substitution was also assessed by evaluating the aggregation of platelet-rich plasma from His^452^/His^452 ^and His^452^/Tyr^452 ^healthy individuals after stimulation with adenosine diphosphate ± serotonin.

**Results:**

The T allele of the C+1354T polymorphism was underrepresented in scleroderma patients compared with control individuals (5.2% versus 12.4%, *P *< 0.001, chi-square test and 1,000-fold permutation test) and its carriage reduced the risk for systemic sclerosis (odds ratio = 0.39, 95% confidence interval = 0.19 to 0.85, *P *< 0.01). Platelets from His^452^/Tyr^452 ^healthy subjects more weakly responded to serotonin stimulation compared with platelets from His^452^/His^452 ^individuals (3.2 ± 2.6-fold versus 9.6 ± 8.6-fold increase in aggregation, *P *= 0.017 by Kolmogorov–Smirnov test and *P *= 0.003 after correction for baseline adenosine diphosphate-induced aggregation values).

**Conclusion:**

The His^452^Tyr substitution may influence susceptibility to systemic sclerosis by altering platelet aggregation in response to serotonin.

## Introduction

Systemic sclerosis (SSc) is a complex connective tissue disease characterised by fibrosis of the skin and internal organs, widespread vasculopathy and abnormalities of the immune system [1]. Whilst the deposition of collagen is the ultimate hallmark of the disease [2], vascular injury and activation are primary events in the pathogenesis of SSc that may sustain the fibrotic process from the earliest phases of the disease [2,3].

Amongst the vascular alterations described in SSc patients, perturbations in platelet homeostasis have long been recognised [4]. Platelets from SSc patients show an activated phenotype [5,6] and highly respond to a variety of aggregating stimuli [7], releasing biomolecules with vasoactive, inflammatory, mitogenic and profibrotic properties [8]. Platelets are a rich source of serotonin (5-hydroxitrpitamine (5-HT)) [9] – a powerful mediator with a wide array of functions, ranging from vasoconstriction in damaged vessels [10], mitogenesis of vascular smooth muscle cells [11] and fibroblasts [12], to the activation of platelets themselves [13,14]. Increased concentrations of 5-HT were found in plasma from SSc patients [15], and the depletion of intraplatelet 5-HT concentrations was also observed in these subjects [16], reflecting both the release of 5-HT from intracellular stores and platelets' persistent activation.

The 5-HT functions are mediated by a superfamily of seven related G-coupled receptors (5-HTR_1 _to 5-HTR_7_) [17], but it is the interaction with the serotonin 2A receptor (5-HTR_2A_) that accounts for most of the detrimental profibrotic, vasoconstrictive, mitogenic and proaggregating activities of 5-HT [10-14]. The 5-HTR_2A _gene is located at 13q14-q21, and several single nucleotide polymorphisms (SNPs) have been identified within this region, a few of which determine amino acid substitutions and may thus be relevant from a functional point of view [18]. Amongst these substitutions, one of the most well characterised is the nonconservative C/T transition at position +1,354 of the third exon of the 5-HTR_2A _gene (C+1354T, rs6314) that determines a His^452^Tyr substitution in the C-terminal region of the receptor [19]. This relatively abundant substitution has variedly been associated with several psychiatric disorders [20] and may influence 5-HT responses by destabilising the intracellular signal, reducing the activation of G-dependent phospholipases C and D [21]. These events may account for the described reduced mobilisation of intracellular calcium of platelets from subjects with the His^452^Tyr substitution [22], and may eventually lead to a blunted platelet aggregation.

In the present study we first explored a possible association between the C+1354T SNP of the 5-HTR_2A _gene and SSc, and we then further clarified its functional role by evaluating platelet aggregation in response to the costimulation with ADP and 5-HT [14] in healthy subjects with either one of the variants of the 5-HTR_2A _gene.

## Materials and methods

### Patient selection

One hundred and fifteen consecutive unrelated Italian SSc patients referred to our outpatient clinic were included. All of the patients fulfilled the classification criteria proposed by the American College of Rheumatology [23], and were categorised as having limited cutaneous SSc or diffuse cutaneous SSc according to LeRoy and colleagues [24]. Disease onset was determined by the patient's recall of the first non-Raynaud symptom clearly attributable to scleroderma [25]. The patients' autoantibody profile was also determined by reviewing the patients' medical records. Antinuclear antibodies were determined by indirect immunofluorescence on Hep_2 _cells (Kallestad, Chaska, MN, USA) using a standardised technique [26]. Extractable nuclear antigens were determined by a commercial ELISA (Diamedix, Miami, FL, USA). The presence of a reduced forced vital capacity (<70% of predicted), of a reduced diffusing capacity for carbon monoxide (<70% of predicted) or of an increased right-ventricular systolic pressure on echo (≥ 40 mmHg) was also assessed.

One hundred and forty healthy ethnically matched, sex-matched and age-matched subjects were also included as control individuals (case-to-control ratio, 1:2).

All of the patients and all of the controls gave their written consent for the present research. The protocols of the study as well as of the functional study described below were approved by the ethic committee of our institution, and are in compliance with the Declaration of Helsinki.

### Sequence-specific primer-PCR for ^452^His/^452^Tyr

Ten millilitres of blood were collected into tubes containing sodium citrate. Genomic DNA was isolated with the DNA Isolation Kit for Mammalian Blood (Roche Diagnostics, Indianapolis, IN, USA). To detect the C+1354T SNP, the 5HTR_2A _gene was amplified using PCR. In brief, a set of primers was designed to encompass the C+1354T polymorphic site in the 5HTR_2A _gene (forward primer, 5'-AGCCAACTTCAAATGGGACA-3' and reverse primer, 5'-CACACACAGCTCACCTTTTCA-3'). The PCR reaction was performed using 100 ng genomic DNA, 10 pM each primer, 1.5 mM MgCl_2_, 2.5 mM dNTPs and 1 U Euro Taq (Euro Clone, Milano, Italy) in a final volume of 25 μl. PCR amplification was initiated at 96°C for 5 minutes and was performed for 40 cycles, each consisting of 30 seconds at 96°C, 45 seconds at 58°C and 45 seconds at 72°C. A final elongation step of 5 minutes at 72°C was added.

### Sequencing

All of the PCR products were sequenced. Prior to sequencing, the unincorporated dNTPs and primer were removed by ExoSAP-IT (USB Corporation, Cleveland, OH, USA) at 37°C for 15 minutes, after which the enzymes were deactivated by incubation at 80°C for 15 minutes. Samples were sequenced in both directions on an Applied Biosystems 3100 Genetic Analyzer using the Big-Dye Terminator Cycle Sequencing Reaction Kit (Applied Biosystems, Foster City, CA, USA). The cycling conditions were 25 cycles of denaturation at 96°C for 10 seconds, annealing at 50°C for 5 seconds and extension at 60°C for 4 minutes.

### Platelet aggregation

Blood samples from ^452^His/^452^Tyr and ^452^Tyr/^452^Tyr healthy nonsmoker individuals were obtained for the functional study; none of these subjects was receiving steroids or antiaggregation therapy. Whole blood, anticoagulated with sodium citrate (final concentration, 3.8%), was immediately centrifuged at 130 × *g *for 15 minutes in order to obtain platelet-rich plasma. A subsequent centrifugation at 1,050 × *g *for 15 minutes allowed one to obtain platelet-poor plasma, used to set the 100% light transmission of the instrument. Then 250 μl platelet-rich plasma was warmed at 37°C for 3 minutes, and the agonist was added. Platelet aggregation was performed on a Chrono-Log Aggregometer (Mascia-Brunelli, Milano, Italy) with ADP (Sigma-Aldrich Corp, Milano, Italy) in a plain Tyrode's solution containing 2 mM CaCl_2_, 1 mM MgCl_2_, 0.1% dextrose, 0.35% BSA, 0.05 U/ml apyrase, pH 7.35, at a final concentration of 1 μM, or with ADP + 5-HT (Sigma-Aldrich Corp.) both at a 1 μM final concentration. Platelet aggregation was recorded for 3 minutes and the maximum light transmission in this period was measured. The response to 5-HT was then calculated as the fold increase in 5-HT-induced aggregation with respect to the aggregation observed after stimulation with ADP alone.

In the present work the decision was made not to replicate the functional study in SSc patients. This decision was mainly due to different reasons. Firstly, all of our patients were being treated with a combination of drugs that may alter platelet function (for example, low-dose aspirin, nifedipine, chronic intravenous iloprost), and thus it would have been unethical to interrupt their ongoing therapy. Secondly, SSc platelets show an activated phenotype that that would have been a significant confounding factor in the analysis of platelet function [5-7].

### Statistical analysis

#### Association study

The distribution of the C+1354T genotypes was tested for Hardy–Weinberg equilibrium with the goodness-of-fit chi-squared test both in patients and in control individuals.

The distribution of the C+1354T genotypes and alleles between control individuals and SSc subjects was tested by the chi-squared test or Fisher's exact test when necessary. Odds ratios and their relative 95% confidence intervals were also calculated from 2 × 2 contingency tables. Statistical significance was also evaluated using a 1,000-fold permutation test.

Genetic-association studies might be flawed by the possibility of false-positive results, even in the presence of statistically significant findings – that is, according to the definition of Wacholder and colleagues [27], the false-positive report probability (FPRP). The FPRP values are calculated by the following formula: FPRP = 1/{1 + [π/(1 - π)] [(1 - β)/α]}, where π is the prior probability that the association is true, α is the type I error probability and β is the type II probability to detect the association under the experimental conditions.

In the present context, α was set to the observed *P *value while π was set from 0.001 up to relatively high values (0.5 – 1), given that only five SNPs within the 5-HTR_2A _gene determine amino acid substitutions and may thus be functionally relevant, and the C+1354T SNP indeed alters platelet function *in vitro *(see reference [22] and the results below).

Finally, the statistical power (1 - β) was calculated by the PS Program [28], and it was defined as the power to detect an odds ratio of 1.5, 1.75 or 2 for the carriers of the ^452^His/^452^Tyr substitution and to detect an odds ratio of 1 for the homozygote with the common variant, with an α level equal to the observed *P *value.

The FPRP values for the present study were then reported in a table with the corresponding π and odds ratio values; FPRP values < 0.5 are then highlighted. These values are considered adequate in small exploratory studies on genetic associations [27] – given that some estimates of the overall FPRP in the molecular epidemiology literature have been up to 0.95 [29].

#### Functional study

The 5-HT-to-ADP aggregation rate in ^452^His/^452^His and ^452^His/^452^Tyr healthy subjects was compared by the nonparametric Kolmogorov–Smirnov test and was then verified by linear regression. As platelet-induced aggregation displays a ceiling effect, the magnitude of the dependent variable (5-HT-to-ADP aggregation ratio) may be correlated with the variance of baseline (ADP-induced) aggregation, thus violating the assumption of homoscedasticity [30]. Linear regression was thus conducted by the weighted least-squares analysis procedure, with the ADP-induced aggregation as the weight variable and with gender as an additional covariate.

All of the statistical procedures were carried out with the SPSS version 15.0 software (SPSS Inc., Chicago, IL, USA). *P *< 0.05 was considered significant.

Continuous values are expressed as the mean ± standard deviation – except for skewed distributions (skewness <-2 or skewness >2), where the median and interquartile range are reported.

## Results

The demographic and clinical characteristics of the patients are reported in Table 1. The genotypes of the C+1354T SNP respected the Hardy–Weinberg equilibrium both in patients and in control individuals. The overall minor allele frequency was 0.092.

**Table 1:** Demographic and clinical characteristics

Variable	Value (*n *= 115)
Females	108 (93.9)
Limited cutaneous systemic sclerosis	84 (73)
Autoantibody	
Antinuclear antibody without specific pattern	19 (16.5)
Anticentromere antibody	44 (38.3)
Antitopoisomerase I antibody	50 (43.5)
Age at onset (years)	43.4 ± 12.6
Mean follow-up (years)	14.6 ± 8.7
Forced viral capacity <70% predicted	27 (23.4)
Diffusing capacity for carbon monoxide <70% predicted	82 (71.4)
Right systolic ventricular pressure ≥ 40 mmHg	22 (19.1)
Oesophageal involvement	110 (96.2)
Past history of digital ulcers	63 (54.7)

Data presented as *n *(%) or as the mean ± standard deviation.

### Association study

As reported in Table 2, the CT genotypes (^452^His/^452^Tyr heterozygosity) and the TT genotypes (^452^Tyr/^452^Tyr homozygosity) of the C+1354T SNP were underrepresented in SSc patients compared with control individuals (χ^2 ^= 7.102, two degrees of freedom, *P *= 0.011). Similarly, a decreased frequency of the T allele was observed in SSc patients (χ^2 ^= 7.308, one degree of freedom, *P *< 0.001). All of the results were confirmed after the 1,000-fold permutation test (*P *< 0.05 and *P *< 0.001 for genotypes and alleles, respectively). Overall, ^452^His/^452^Tyr and ^452^Tyr/^452^Tyr individuals had a threefold reduction in the risk for SSc compared with ^452^His/^452^His individuals (odds ratio = 0.39, 95% confidence interval = 0.19 to 0.85, *P *< 0.01).

**Table 2:** Genotype and allele distribution of the C+1354T single nucleotide polymorphism in patients with systemic sclerosis and in matched control individuals

C+1354T single nucleotide polymorphism	Systemic sclerosis patients (*n* = 115)	Control individuals (*n* = 140)
Genotype*		
CC	103 (89.6)	108 (77.1)
CT	12 (10.4)	30 (21.4)
TT	0 (0)	2 (1.4)
Allele**		
C	218 (94.8)	246 (87.6)
T	12 (5.2)	34 (12.4)

Data presented as *n *(%).*P < 0.05. **P < 0.01 (chi-square test).

The FPRP values of the association between SSc and the ^452^His/^452^Tyr substitution are reported in Table 3 with highlighted noteworthiness values at the 0.5 level.

**Table 3:** False-positive report probabilities under different scenarios

Prior probability	Odds ratio		
	
	1.5	1.75	2
0.1	*0.48*	*0.29*	*0.19*
0.05	0.66	*0.46*	*0.33*
0.01	0.91	0.82	0.72
0.001	0.99	0.98	0.97

False-positive report probability values are presented for the association between the C+1354T single nucleotide polymorphism and systemic sclerosis in our population. Different ranges of values are reported in relation to the prior probability values and the power to detect different odds ratios for ^452^His/^452^Tyr individuals compared with ^452^His/452His subjects, with α = 0.009. Data in italics are reported noteworthy false-positive report probability values at the 0.5 level.

Robust associations with clinical variables were difficult to calculate owing to the low prevalence of the rarer allele of the C+1354T SNP in SSc patients; however, from an exploratory point of view, the His^452^Tyr substitution was not associated with any of the following: the disease subset, the anticorpal status, age at the onset of the disease, past history of digital ulcers, the forced viral capacity, the diffusing capacity for carbon monoxide or the right systolic ventricular pressure.

### Platelet aggregation

**Figure 1:**
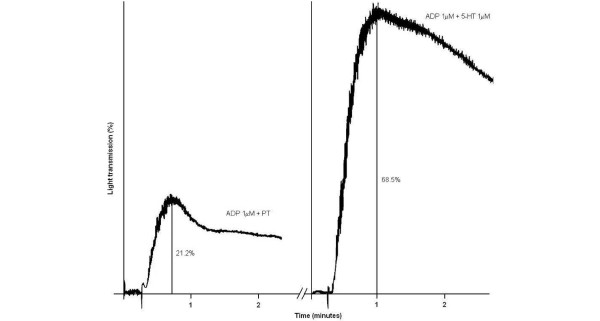
Platelet aggregation induced by ADP and serotonin. Left panel: aggregation induced by 1 μM ADP + 1 μM buffer solution (plain Tyrode's solution (PT)). Right panel: aggregation induced by 1 μM ADP + 1 μM serotonin (5-HT). The ratio between the two highest light transmission percentages is the 5-HT-to-ADP response ratio (in the example, 68.5/21.2 = 3.23-fold increase).

Platelets from 15 healthy subjects (eight ^452^His/^452^His homozygous subjects, seven ^452^His/^452^Tyr heterozygous subjects) had poor aggregating responses after stimulation with ADP alone (median, 11.8%; interquartile range, 5.4% to 25% light transmission). Aggregating responses were markedly increased after costimulation with 5-HT and ADP (median, 3.8-fold increase; interquartile range, 2.9-fold to 8.6-fold) with respect to the stimulation observed with ADP alone (Figure 1). These data are consistent with previous studies reported in the literature [14].

**Figure 2:**
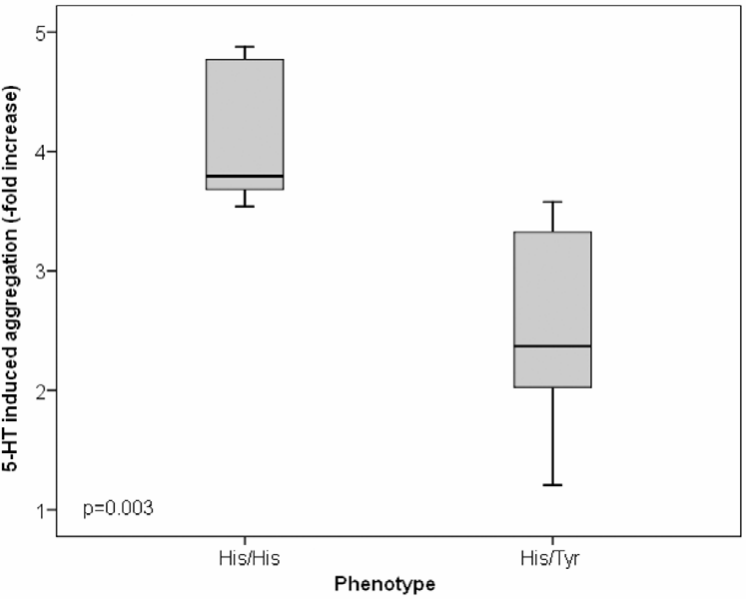
Serotonin-induced platelet aggregation in subjects with the ^452^His/^452^His or the ^452^His/^452^Tyr phenotype. Fold increase of serotonin (5-HT)-induced platelet aggregation after correction for baseline ADP aggregation values by the weighted least-squares analysis procedure (see Materials and methods) in subjects with the ^452^His/^452^His or the ^452^His/^452^Tyr phenotype. Black centre line, median for each dataset; boxes, interquartile range; bars, cases (three box-lengths form the 25th or the 75th percentile).

The ADP-induced baseline aggregation, age, platelet count and gender distribution did not differ between ^452^His/^452^His individuals and ^452^His/^452^Tyr individuals. The ^452^His/^452^Tyr heterozygous subjects had blunted responses to 5-HT stimulation compared with ^452^His/^452^His homozygous subjects (mean, 3.2 ± 2.6-fold versus 9.6 ± 8.6-fold increase, *P *= 0.017). The functional significance of the His^452^Tyr substitution was better assessed by linear regression analysis, weighted for ADP-induced platelet aggregation; by this procedure, it was confirmed at a highly significant level that the His^452^Tyr substitution of the 5-HTR_2A _dampened platelet aggregation in response to 5-HT (Figure 2, *P *= 0.003). The regression equation of the final model with adjusted *R*^2 ^= 0.98 had an almost perfect fit: 5-HT-to-ADP-response ratio (fold increase) = 4.995 - (0.019 × ADP-induced aggregation) - (1.148 × gender) - (1.192 × His^452^Tyr substitution) (where zero represents females and/or ^452^His, and one represents males and/or ^452^Tyr).

## Discussion

Owing to the evidence of involvement of vasculopathy in the pathogenesis of SSc [2,3], much research has been carried out to elucidate the role of genetic variants of biomolecules with vascular activities in scleroderma patients [31-37]. Despite the number of studies indicating a role for platelets and the 5-HT system in the onset of or in the maintenance of the vascular damage in SSc [4], however, no genetic research has so far been conducted in this field. The present study was thus undertaken to analyse, for the first time, the distribution and the functional role of a naturally occurring amino acidic substitution of the 5-HTR_2A _gene in a population of Italian SSc patients.

Our results indicate that the C/T transition at position +1,354 of the third exon of the 5-HTR_2A _gene [19] is associated with a threefold reduction in the risk for SSc. This effect could be linked to a blunted platelet aggregation in response to the serotoninergic stimulus in ^452^His/^452^Tyr heterozygotes compared with ^452^His/^452^His homozygotes, as indicated by our *ex vivo *study (Figure 2). This observation is in full accordance with the finding that the His^452^tyr substitution in the 5-HTR_2A _gene is accompanied by a reduced mobilisation of platelet intracellular calcium after stimulation with 5-HT [22].

The 5-HT concentrations are increased in plasma samples from SSc [16] as a consequence of platelet activation that follows the binding to collagen type I and type III exposed under the damaged endothelium, which is also favoured by T-dependent immunological mechanisms [4]. It is thus possible to speculate that the carriage of the C+1354T SNP dampens the mechanisms that sustain platelet aggregation and SSc vasculopathy [2,4], once they have been triggered via other biological pathways. This hypothesis would confirm previous findings indicating that 5-HT is more relevant in the maintenance of the vascular phenomena that underlie the pathogenesis of SSc, rather than in determining their onset [38]. We cannot, however, exclude the His^452^Tyr substitution possibly having a role in SSc susceptibility by acting on different cellular types that express the 5-HTR_2A _gene (for example, fibroblasts or vascular smooth muscle cells), regardless of its effect on 5-HT-induced platelet aggregation. Indeed, Hazelwood and Sanders-Bush also described a reduced intracellular signalling capacity in murine fibroblasts expressing the His^452^Tyr substitution after stimulation with 5-HT [21].

The potential limitations of our study are the relatively small sample size and the lack of a replicate population. We feel confident that the evidence demonstrating the functional relevance of the His^452^Tyr substitution, however, makes our findings modestly prone to false positive results. The significance of our results can also be gauged considering the FPRP values we obtained under different scenarios (Table 3). Whilst no significant FPRP values were observed for prior probabilities ≤ 0.01, which were originally advocated as an adequate setting for a gene with functional data [27], it may be argued that these prior probability values, or conversely the FPRP 0.5 threshold, may be too penalising for an exploratory study such as ours. Even if no single point mutation is therefore likely to determine the onset of a multifactorial disease such as SSc, our results indicate that the C+1354T SNP is a suitable SNP for further research in the scleroderma field and that it may be worthy of inclusion in association studies based on a candidate gene approach [39]. Of particular interest would also be the study of epistatic interactions or intermediate quantitative trait analysis between this mutation and other genetic variants of the 5-HT_2A _gene or other serotonin receptors, such as the 5-HT_3A _gene that was also found to play a role in the fibrotic process of SSc [40,41]. Finally, the demonstration that the C+1354T SNP is indeed associated with a reduced platelet aggregation after stimulation with 5-HT may have practical implications besides SSc – that is, in other diseases where the serotoninergic system is involved.

## Conclusion

We provide evidence that the His^452^Tyr substitution of the 5-HT_2A_receptor, determined by the C+1354T SNP of the corresponding gene, is functionally relevant to platelet aggregation *in vitro*, dampening the responses to the serotoninergic stimulus. The functional relevance of this polymorphism may explain the inverse association (for example, protective effect) we observed in a population of Italian SSc patients. The C+1354T SNP is therefore worth inclusion in a panel of candidate genes for future association studies in the SSc field.

## Abbreviations

BSA: bovine serum albumin; ELISA: enzyme-linked immunosorbent assay; FPRP: false-positive report probability; 5-HT: serotonin; 5-HTR_2A_: serotonin 2A receptor; PCR: polymerase chain reaction; SSc: systemic sclerosis; SNP: single nucleotide polymorphism.

## Competing interests

The authors declare that they have no competing interests.

## Authors' contributions

LB was responsible for the study design, manuscript preparation, analysis and interpretation of data, and statistical analysis. MC participated in collection of data, interpretation of data and genetic analysis. MM performed genetic analysis. FC was responsible for manuscript preparation and interpretation of data. AA and GM performed the platelet functional study. RS was responsible for the study design and fundraising.
